# Effects of Climate and Rodent Factors on Hemorrhagic Fever with Renal Syndrome in Chongqing, China, 1997–2008

**DOI:** 10.1371/journal.pone.0133218

**Published:** 2015-07-20

**Authors:** Yuntao Bai, Zhiguang Xu, Bo Lu, Qinghua Sun, Wenge Tang, Xiaobo Liu, Weizhong Yang, Xinyi Xu, Qiyong Liu

**Affiliations:** 1 Division of Environmental Health Sciences, College of Public Health, The Ohio State University, Columbus, Ohio, United States of America; 2 Department of Statistics, The Ohio State University, Columbus, Ohio, United States of America; 3 Division of Biostatistics, College of Public Health, The Ohio State University, Columbus, Ohio, United States of America; 4 Chongqing Center for Disease Control and Prevention, Chongqing, China; 5 Key Laboratory of Surveillance and Early-Warning on Infectious Disease, Chinese Center for Disease Control and Prevention, Beijing, China; 6 State Key Laboratory for Infectious Disease Prevention and Control, Collaborative Innovation Center for Diagnosis and Treatment of Infectious Diseases, National Institute for Communicable Disease Control and Prevention, Chinese Center for Disease Control and Prevention, Beijing, China; University of Texas Medical Branch, UNITED STATES

## Abstract

China has the highest global incidence of hemorrhagic fever with renal syndrome (HFRS), constituting 90% of the cases in the world. Chongqing, located in the Three Gorges Reservoir Region, has been experiencing differences in the occurrence of HFRS from 1997 to 2008. The current study was designed to explore the effects of climate and rodent factors on the transmission of HFRS in Chongqing. Data on monthly HFRS cases, rodent strains, and climatic factors were collected from 1997 to 2008. Spatio-temporal analysis indicated that most HFRS cases were clustered in central Chongqing and that the incidence of HFRS decreased from 1997 to 2008. Poisson regression models showed that temperature (with lagged months of 0 and 5) and rainfall (with 2 lagged months) were key climatic factors contributing to the transmission of HFRS. A zero-inflated negative binomial model revealed that rodent density was also significantly associated with the occurrence of HFRS in the Changshou district. The monthly trend in HFRS incidence was positively associated with rodent density and rainfall and negatively associated with temperature. Possible mechanisms are proposed through which construction of the dam influenced the incidence of HFRS in Chongqing. The findings of this study may contribute to the development of early warning systems for the control and prevention of HFRS in the Three Gorges Reservoir Region.

## Introduction

Hemorrhagic fever with renal syndrome (HFRS), a rodent-borne disease caused by hantaviruses, is characterized by fever, acute renal dysfunction, and hemorrhagic manifestations [1]. HFRS is primarily found throughout the Asian and European continents [2]. In Europe, the most important hantavirus is Puumala virus [3–5], while in China there are two main serotypes of hantaviruses: Hantaan virus and Seoul virus [6]. HFRS, caused by Hantaan virus, occurs year-round, but tends to peak in the winter while HFRS cases associated with Seoul virus typically peak in the spring [7]. In addition, a novel hantavirus (Amur virus) has been identified in some areas of China [8].

China is the most severe endemic country with 90% the total HFRS cases in the world. During the 58-year period of 1950–2007, a total of 1,557,622 HFRS cases were reported in China. The annual incidence of HFRS in China peaked in 1986 at 11.08/100,000 and declined in the late 1990s [9]. From 2000 to 2007, the annual number of HFRS cases declined >3-fold, from 37,814 to 11,248 because of multiple intervention measures, such as rodent control, vaccination, and environment management [10]. However, HFRS remains a serious public health threat in some areas of China [9, 11, 12].

The hantaviruses are transmitted to humans through various rodent strains that serve as natural hosts. Humans usually are infected with hantaviruses through contact with, or inhalation of, aerosols or secretions from infected rodents [12]. Each hantavirus has co-evolved with a distinct rodent host; e.g., Hantaan virus is mainly carried by the striped wild mouse (*Apodemus agrarius*), whereas Seoul virus is mainly carried by domestic rat (*Rattus norvegicus*) in China [13].

Previous studies have suggested that socioenvironmental factors influence the incidence of HFRS, such as social-economic circumstances (e.g. resident income, education, occupation and relocation), climate fluctuation, and geological heterogeneity [14, 15]. Climatic factors contributing to HFRS transmission include: relative humidity of the air, moisture of the soil, temperature, and rainfall [4, 16–18]. These climatic factors may trigger variations in HFRS incidence, not only having a role in breeding and survival of rodent populations, but also on the transmission of hantaviruses [9].

Chongqing is located in the Three Gorges Reservoir Region, which has been experiencing vast natural and socioeconomic changes because of the construction of the Three Gorges Dam (TGD); the world’s largest hydroelectric facility. A previous study indicated that some areas in Chongqing were medium endemic areas with HFRS incidences between 5.0/100,000 and 30.0/100,000 from 1994 to 1998 [15]. However, few studies have been conducted to explore the dynamics of HFRS occurrence and determine the potential spatio-temporal factors leading to this disease in Chongqing after this period. In the present study, the spatio-temporal patterns of HFRS case distributions were explored and key climatic drivers of HFRS transmission were identified in Chongqing from 1997 to 2008.

## Methods

### Study sites and data collection

**Fig 1:**
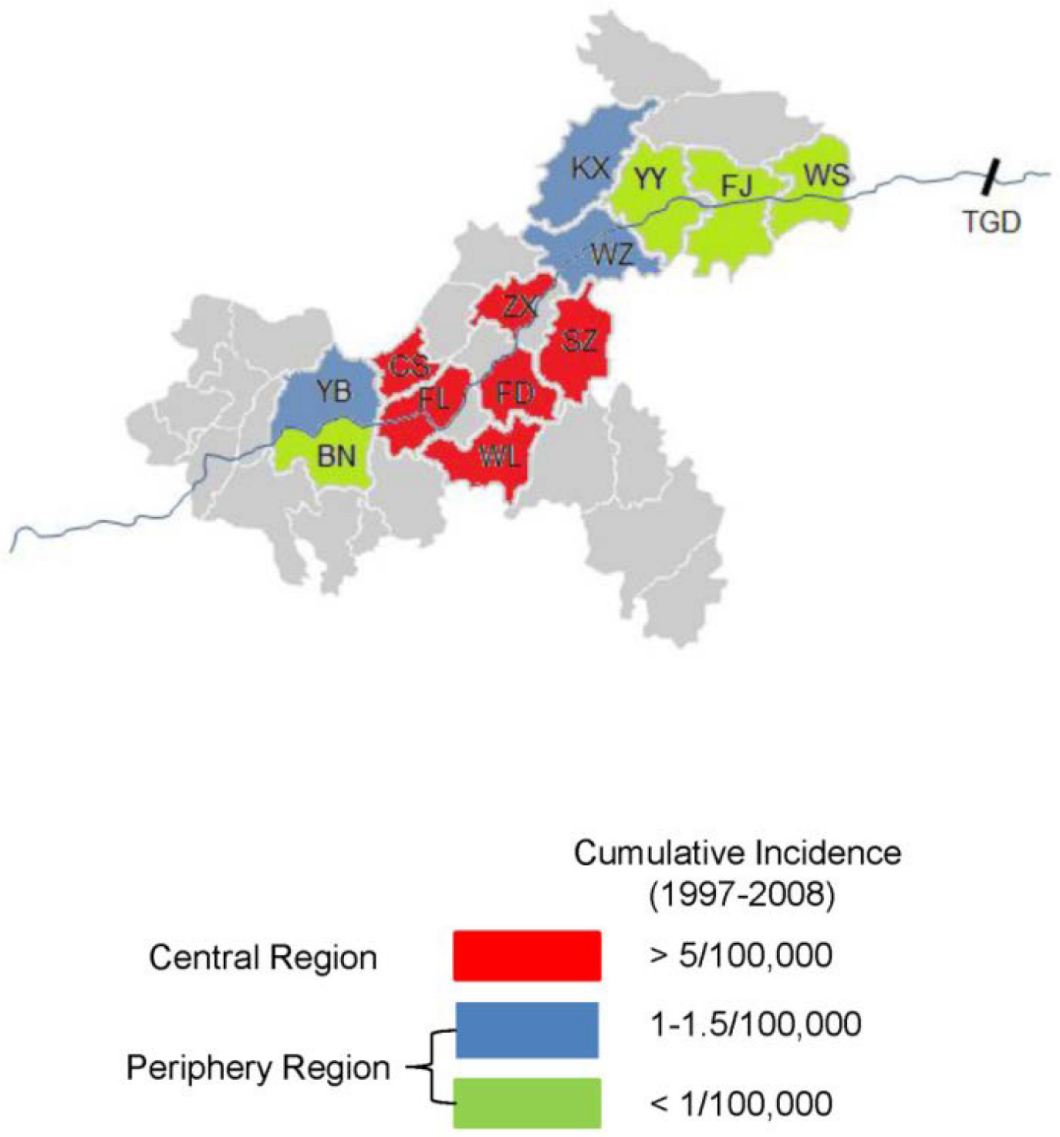
Spatial distribution of HFRS occurrence from 1997 to 2008. WS: Wushan; FJ: Fengjie; YY: Yunyang; KX: Kaixian; WZ: Wanzhou; SZ: Shizhu; ZX: Zhongxian; FD: Fengdu; WL: Wulong; FL: Fuling; CS: Changshou; YB: Yubei; BN: Banan. TGD: Three Gorges Dam. HFRS: hemorrhagic fever with renal syndrome. Central region includes the districts of ZX, CS, SZ, FL, FD, and WL. The rest of districts are designated as periphery region.

Study sites and monthly meteorological data collection have previously been described [19].The sites were composed of 13 districts: Banan, Changshou, Fengdu, Fengjie, Fuling, Kaixian, Wanzhou, Shizhu, Wulong, Wushan, Yubei, Yunyang and Zhongxian. These districts are located along the Yangtze River (Fig 1).

The case of HFRS was selected according to epidemiological data, clinical symptoms and signs. Patient blood samples were collected and sent to local Center for Disease Control and Prevention (CDC) institutes for serological and etiological confirmation. All cases were confirmed on antibody tests, pathogen isolation, or evidence of hantavirus RNA sequences in blood or tissues. Patient data used in this study were analyzed and reported anonymously. All participants provided their written informed consent to participate in this study; and ethical approval for this study was obtained from the Ethical Review Committee of China CDC (No: 201214).

Multiple processes were conducted to control the data quality during HFRS surveillance. First, surveillance system and training guidelines were followed by local CDC employees during data collection and analysis; any abnormal values were confirmed by either the local CDC or China CDC. Second, reported cases were regularly reviewed to guarantee data integrity. Third, physicians were required to report HFRS cases to the local CDC within 12 hours of the occurrence according to laws and regulations.

Rodent data were collected every April and September from 1997 to 2007 in residential area and field areas in the Changshou district. The residential area was selected based on the representative of ecological habitats and the occurrence of HFRS in this district. In residential area, 150 mouse traps were placed daily for a month and effective traps must be higher than 130 traps. One or two traps were placed in a room based on the areas, and five traps were placed in a household for 30 houses. For the field area, forestry and farmland were selected to conduct the investigation of rodent density; 150 traps were placed per habitat daily for a month. To capture the rodents, mouse traps were set at 5-meter intervals and baited with peanuts. The captured rodents were taxonomically identified to strain level according to criteria developed by Chen and Qiu [20]. Rodent density was calculated as a proportion (total number of captured rodents/total number of valid mouse traps). An invalid mouse trap was defined as either a missing trap or non-rodent triggered trap.

### Spatio-temporal analysis of HFRS incidence

Cumulative incidences (total number of cases/population at the beginning of study) were calculated for each district to explore the spatial trend of HFRS. The annual/monthly HFRS incidences were calculated for the study area between 1997 and 2008 and curves were plotted to explore the temporal pattern. The number of cases occurring in each month was presented as mean ± standard error of mean to determine the seasonal pattern of HFRS.

### Statistical model

Poisson regression models were used to explore the association between HFRS incidence and climatic factors. The autocorrelation of incidence was examined and noticeable autocorrelation with lags of 1, 3, and 6 months were demonstrated. In these two plots, we found that the lag-1, lag-3 and lag-6 autocorrelations were 0.573, 0.458 and 0.512, respectively, which were significant under the level 0.05 (the minimum absolute value for autocorrelation coefficient to be significant under 0.05 with the sample size of 140 was calculated to be Φ^−1^((1+0.95)/2)/$\sqrt{140}=$ 0.166, in which Φ^−1^ is the inverse function of cumulative distribution function of standard normal distribution); their partial autocorrelation were 0.573, 0.279 and 0.220, which were larger than the other lags up to 12. Additionally, the AIC values increased when more autocorrelation terms were added to the model. Therefore, the incidence with lags of 1, 3 and 6 was incorporated into our models as predictors to account for the autocorrelation.

Annual gross domestic product (GDP) values for Chongqing were included as a predictor to control for the impact of economic improvement on HFRS incidence. A preliminary analysis was conducted using the Poisson regression model with incidence (lags of 1, 3 and 6 months) and GDP values as predictors; y_t_ denotes the count of HFRS at time t. According to the Poisson regression model, y_t_ followed a Poisson distribution with mean μ_t_. We denoted the incidence at time t as rate_t_ = μ_t_/N_t_, where N_t_ denotes the population of a district at time t, the incidence with lag 1, 3 and 6 months as rate_t-1_, rate_t-3_ and rate_t-6_, and GDP value at time t as gdp_t_. The preliminary analysis model was:

$$Log(rate_{t})=\zeta_{0}+\delta_{1}\times rate_{t-1}+\delta_{2}\times rate_{t-3}+\delta_{3}\times rate_{t-6}+u_{2}\times gdp_{t}$$

Where δ_1_, δ_2_, δ_3_, and u_2_ are the corresponding regression coefficients before these predictors. Following the preliminary analysis, the association between HFRS incidence and climatic factors was explored with the incidence’s autocorrelation and GDP being adjusted. The multi-collinearity of the different climatic factors with different lags was checked first, then temperature and rainfall data with lags as our candidate predictors were included, based on the multi-collinearity checking. According to the selection process, the final model with the following predictors was constructed: incidence with lag 1, 3 and 6 months (rate_t-1_, rate_t-3_ and rate_t-6_); GDP with lag 0 months (gdp_t_); temperature with lag 0 and 5 months (denoted as temp_t_, temp_t-5_) and rainfall with lag 2 (denoted as rain_t-2_). The model is presented here:

$$\begin{array}{l} Log(rate_{t})=\zeta_{0}+\delta_{1}\times rate_{t-1}+\delta_{2}\times rate_{t-3}+\delta_{3}\times rate_{t-6}+\beta_{21}\times temp_{t}\\ +\beta_{22}\times temp_{t-5}+\gamma_{21}\times rain_{t-2}+u_{2}\times gdp_{t} \end{array}$$

Autocorrelation plot and residual plot of the residuals were used to examine appropriateness of the models.

A zero-inflated negative binomial model was used to explore the relationship between the HFRS incidence and rodent density. Since the number of mice was only collected in April and September in the Changshou district, we aggregated the monthly HFRS incidences into two periods with the same number of months: March-August, and September- February. This aggregation provided more counts of HFRS for each period, which led to more reliable regression analysis. Because the rodent density may impact the disease incidence with lags, rodent density of lags 0 and 1 (denoted as mice_t_, mice_t-1_) were used as our predictors: where y_t_ denotes the count of HFRS at time t, and rate_t_ = μ_t_ /N_t_. The zero-inflated negative binomial model assumed that y_t_ = 0 with probability p_t,_ and y_t_ followed a negative binomial distribution with mean μ_t_ with probability 1-p_t_. The following model was formulated:

$$Logit(p_{t})=\mu_{0}+\alpha_{1}\times mice_{t}+\alpha_{2}\times mice_{t-1}$$

$$Log(rate_{t})=\zeta_{0}+\delta_{1}\times mice_{t}+\delta_{2}\times mice_{t-1}$$

We used R to estimate the models with the function “glm”, which used the iteratively weighted least squares method in order to fit the model and get the parameter estimation. We quantified the significance of parameters with an (asymptotic) z-test. Also, we studied model fitting with log-likelihood (or equivalently, deviance) and examine basic model assumptions with model diagnostic plots.

## Results

### Spatio-temporal patterns of HFRS occurrence

**Fig 2:**
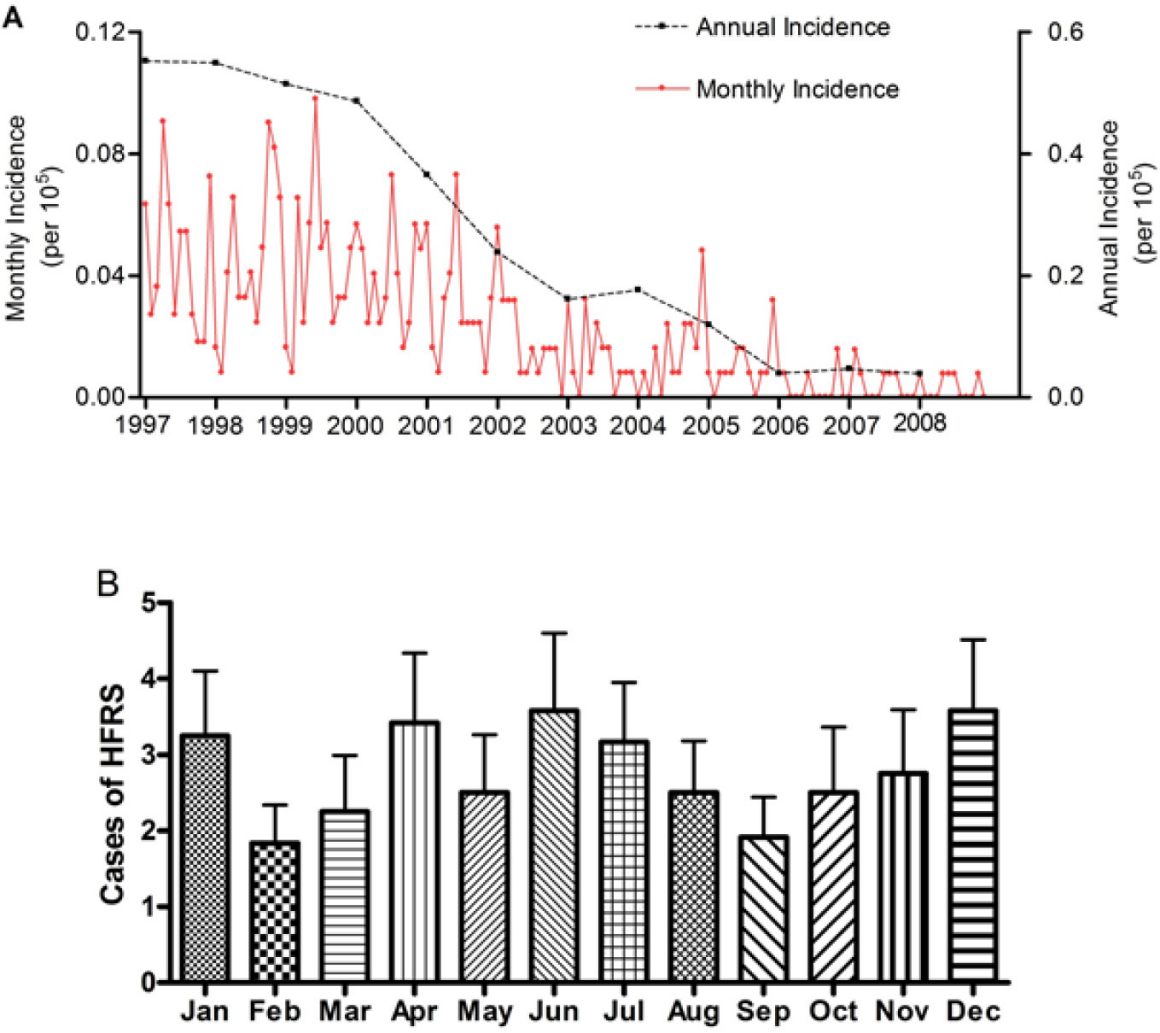
Temporal pattern of HFRS occurrence from 1997 to 2008. (A) Annual and monthly trends of HFRS incidence in the study area; (B) Seasonal trend of HFRS incidence in the study area. HFRS: hemorrhagic fever with renal syndrome. Case numbers for each month are presented as mean ± standard error of mean.

Between 1997 and 2008, a total of 399 cases of HFRS were reported in the study area, with the cumulative number of HFRS cases in each district ranging from 1 to 83. Interestingly, about 85% of the total cases and high cumulative incidences (>5/100,000) occurred in the central region, indicating a spatial trend of HFRS occurrence (Fig 1). In the study area, the annual HFRS incidence decreased from 0.55/100,000 in 1997 to 0.03/100,000 in 2008 (Fig 2A); HFRS cases occurred year-round and tended to peak in April, June and December (Fig 2B).

### Comparison of climatic factors between central and periphery regions

**Fig 3:**
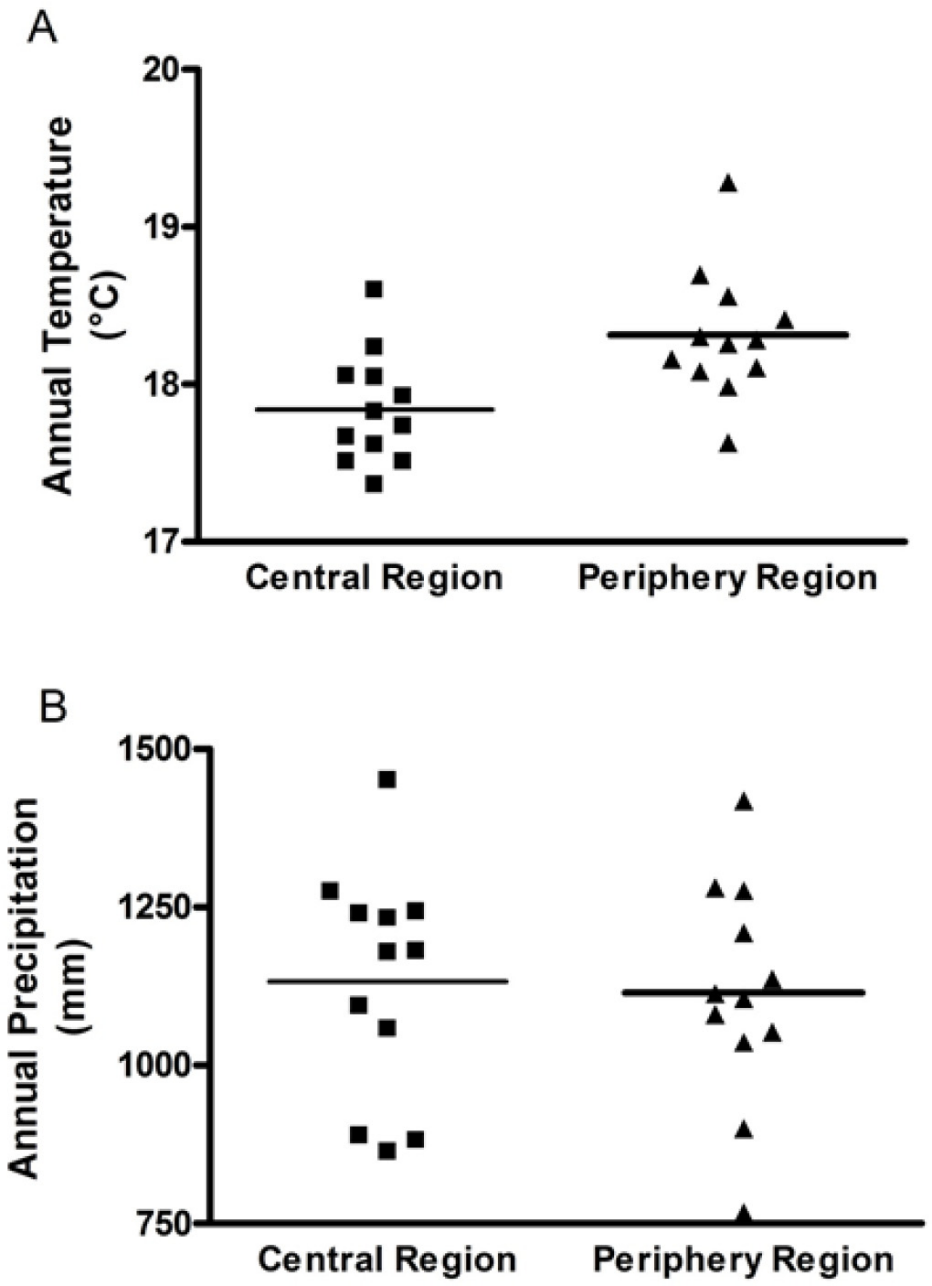
Comparison of climatic factors between central and periphery regions from 1997 to 2008. (A) Difference in annual temperatures between central and periphery regions; (B) Difference in annual precipitations between central and periphery regions.

In the central region, the annual average temperature was 17.8°C and ranged between 17.3°C and 18.6°C (Fig 3A). In the periphery region, the annual average temperature was 18.3°C and ranged between 17.6°C and 19.4°C (Fig 3A). The annual precipitation in the central region was 1132 mm and ranged from 863 to 1450 mm (Fig 3B). In the periphery region, the annual precipitation was 1117 mm and ranged from 746 mm to 1423 mm (Fig 3B).

### Association between climatic factors and HFRS incidence

The difference of the deviance between null model and the fitted model was 203.71, which was significant under level 0.05 (exceeding the upper 5^th^ quantile of chi-square distribution with d.f. = 7), demonstrating that the fitted model was significant under this level.

Based on the preliminary Poisson regression model fitting, GDP was negatively associated with the incidence of HFRS in the study area; GDP would need to be included as a predictor in the final model. The fit of Poisson regression model with climatic predictors showed that temperatures (with a lag of 0 and 5 months) were negatively associated with disease incidence (Table 1). It also showed that rainfall (with a lag of 2 months) was positively associated with incidence of HFRS in the study area (Table 1).

**Table 1:** Association between climatic factors and HFRS incidence in Chongqing City from 1997 to 2008, analyzed by Poisson regression models.

Covariate	Coefficient	95% confidence interval	p value
**Temperature (°C)**	L0 = -0.0459	(-0.085, -0.007)	0.020
	L5 = -0.0392	(-0.073, -0.005)	0.023
**Rainfall (mm)**	L2 = 0.0028	(0.001, 0.005)	0.007
**GDP ($)**	L0 = -0.0044	(-0.006, -0.003)	<0.001

HFRS: hemorrhagic fever with renal syndrome; GDP: gross domestic product; Lx: the lagged months.

With temperature, rain and GDP incorporated in the model, the lag-1, lag-3 and lag-6 autoregressive coefficients were 7.4169, 1.1779 and 4.5365, with p-values being 0.0039, 0.6500, and 0.0701, respectively. It indicated that the lag 1 and 6 autocorrelations were marginally significant under level 0.1; lag 3 autocorrelation, however, was not significant. The residual of the fitted model was approximately symmetric around zero without systemic patterns and normally distributed, which demonstrated few evidence of autocorrelation (S1 Fig).

### Association between rodent density and HFRS incidence

**Fig 4:**
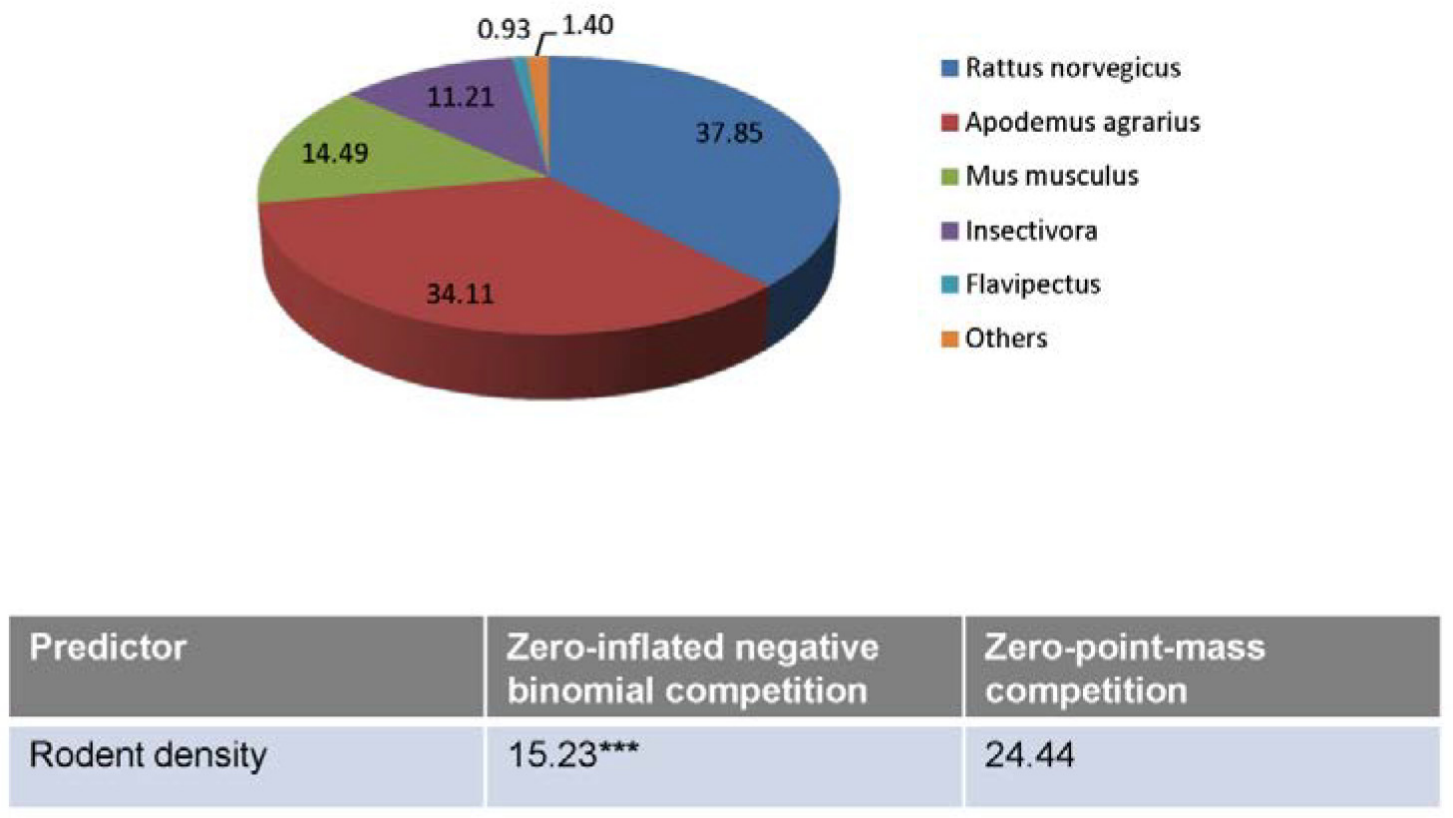
Rodent strain and zero-inflated model in the Changshou district from 1997 to 2007. Zero-inflated model showed that the coefficient of rodent density was significantly positive in the negative binomial regression part but not significant in the zero inflation (logistic regression) part, indicating an overall positive association between the HFRS incidence and rodent density.

Given the limited data, this analysis was only conducted for the Changshou district from 1997 to 2007. The results showed that *Apodemus agrarius* and *Rattus norvegicus* (34.11% and 37.85% of the total rodents) were the main rodent strains; they are the natural hosts for Hantaan and Seoul virus, respectively. Other rodent strains found in this district included *Mus musculus*, *Insectivora*, and *Flavipectus* (Fig 4). The zero-inflated model showed that the coefficient of rodent density with lag 0 was significant in the negative binomial regression part, but not significant in the zero inflation (logistic regression) part (Fig 4), suggesting an overall positive association between the HFRS incidence and rodent density with lag 0.

## Discussion

In the current study, spatio-temporal analysis indicated that most of HFRS cases were clustered in central Chongqing; the incidence of HFRS decreased from 1997 to 2008. Poisson regression models revealed that temperature (with lagged months of 0 and 5) and rainfall (with 2 lagged months) were key climatic factors contributing to the transmission of HFRS. In addition, rodent density was positively associated with HFRS incidence in the Changshou district from 1997 to 2007.

A previous study showed that some districts of Chongqing were medium HFRS endemic areas from 1994 to 1998 [15]; whereas our data revealed that all of 13 districts selected in this study had low HFRS incidences (0–1.5/100,000) from 1997 to 2008. However, 84.71% of the total cases and high cumulative incidences (>5/100,000) occurred in the central region, indicating a spatial trend of HFRS occurrence. Despite the dynamic changes in Chongqing, there was a significant decrease in the incidence of HFRS from 1997 to 2008. Multiple factors may have contributed to spatio-temporal trends of HFRS involving climatic factors, rodent density, and anthropogenic factors.

It has been shown that temperature, moisture, and rainfall are crucial to hantavirus incubation and transmission [4, 18, 21]. Temperature may affect rodent dynamics and activity, as well as transmission of hantavirus. Our data show that there was a negative association between temperature (with lags of 0 and 5 months) and HFRS incidence in Chongqing, which was supported by the findings of previous reports [11, 18]. Studies have suggested that the breeding rate of rodents is highest at temperature of 10–25°C and that a favorable condition for outdoor activity and work is 17°C [22]. Chongqing is located in a subtropical monsoon climate zone and throughout the year has seven months during which the average temperature is over 17°C. High temperature may cause the decrease in rodent density, which contributes to the decrease in HFRS incidence. In addition, high temperature may limit the time for outdoor activity and work, thereby reducing the opportunity for contact between people and the striped field mouse (*A*. *agrarius*), which is one of the most common agricultural pests and a natural vector of hantavirus [23, 24]. However, inconsistent findings have been reported in other studies indicating a positive association between temperature and HFRS incidence [25, 26]. This discrepancy might be due to differences in the environment and climate in the study regions, differences in rodent composition, or variation in the serotypes of hantavirus.

Our data indicate that rainfall with lag 2 was positively associated with the incidence of HFRS; this finding is consistent with most of previous studies [22, 27, 28]. Abundant precipitation can have significant impacts on the transmission of hantaviruses by increasing the growth of vegetation that either directly or indirectly provides food for rodent hosts [6]. Furthermore, moist soil provides suitable conditions for breeding and subsequent increases in rodent populations [29, 30]. However, a recent study showed divergent findings [5], indicating that the impacts of rainfall on HFRS incidence may be more complicated and dependent on the local environment.

In the current study, the HFRS cases occurred year-round, but tended to peak in April, June, and December between 1997 and 2008. Seasonal patterns of HFRS are related to varying transmission dynamics of the major subtypes of hantaviruses. It has been shown that HFRS, caused by Hantaan virus, occurs year-round, but tends to peak in fall and winter, while HFRS caused by Seoul virus typically peaks in spring. These viruses are mainly transmitted by *A*. *agrarius* and *R*. *norvegicus* strains, respectively [7, 13]. Considering the seasonal pattern of HFRS, we infer that HFRS in Chongqing is transmitted by both rodent strains, which is supported by data demonstrating the presence of both in the Changshou district.

**Fig 5:**
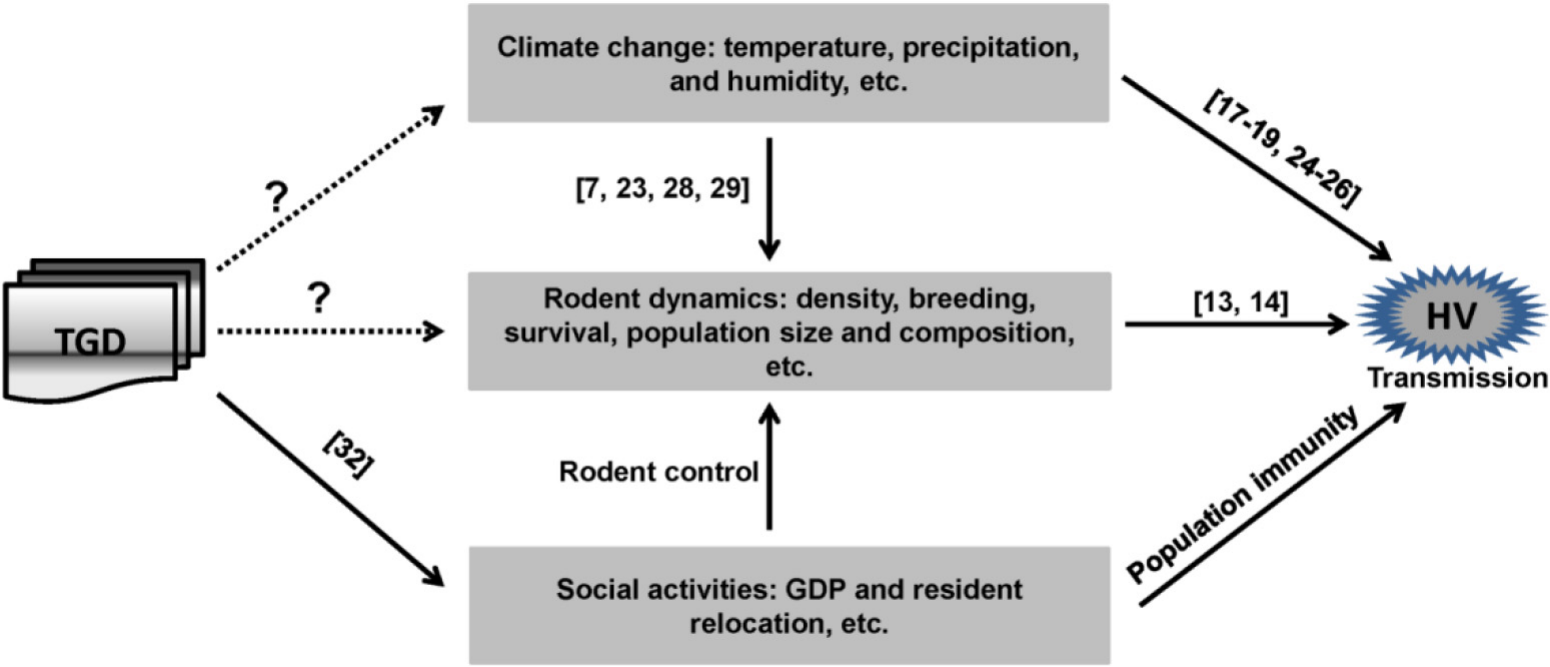
Proposed mechanisms of TGD-associated HFRS occurrence. The occurrence of HFRS involves multiple factors including climate factors, rodent factors and social activities. Climate factors and social activities can affect hantavirus transmission directly or indirectly through changing rodent dynamics. Although the effects of dam construction on the social activities have been reported, whether the dam affects HFRS occurrence through changing local climate and/or rodent dynamics remains elusive. TGD: Three Gorges Dam. HFRS: hemorrhagic fever with renal syndrome. HV: hantavirus. GDP: gross domestic product.

Although the current data are not sufficient to support a direct impact of the TGD on transmission of HFRS, we may propose several scenarios (Fig 5). The construction of the TGD (1997–2012) has resulted in significant ecological changes and substantial modifications to the depth and flow pattern of the river [31–33]. In addition, about 1.24 million residents have been relocated during dam construction [29]. The subsequent changes in the Yangtze River may influence the local rodent reservoir, breeding habits, and population size. Resident relocation and rodent dynamics may increase or decrease the contact between residents and infected rodents, thereby influencing the incidence of HFRS. In our study, a positive association was obtained between rodent density and HFRS incidence in the Changshou district. However, further investigations are needed to resolve the impact that the TGD will have on the rodent dynamics and transmission of HFRS.

TGD may also affect the transmission of hantavirus through changes in local conditions. An important function of the dam is to reduce the frequency of major downstream flooding. However, significant changes in the amount of water, upstream and downstream of the dam, may induce local climate changes. In a previous study, we reported seasonal fluctuations in climatic factors in the divided regions of Chongqing from 1997 to 2008 [19]. However, because pre- and post-construction data were not available, this study could not reach any conclusions concerning TGD construction and local climate changes; long-term data collection will be required to verify association between the TGD and local climatic conditions.

There were some limitations in the current study. First, although the quality of data on HFRS cases was well-monitored, under-reporting and misreporting might still be possible in the disease surveillance system. But it seems unlikely that small changes in HFRS incidence reporting would make a significant change in the general conclusions from our results. Second, our model did not include other potential confounding factors that might have affected the incidence data for HFRS; e.g., resident relocation, socioeconomic status, and level of immunity. Finally, the temperature used in our models was the average temperature; the effects of extreme temperatures on the survival and reproduction of rodents and transmission of HFRS require further investigation.

Climate and rodent factors in the next years should be considered as early warning indicators for HFRS breaks in Chongqing City. The relationship between climate factors and HFRS incidence is particularly valuable since local climate in our study area has changed over the past 12 years [19]. Further investigation, based on long-term data collection, is required to determine if the TGD may affect HFRS occurrence in Chongqing. Such data would be beneficial for control of HFRS in the Three Gorges Reservoir Region.

## Supporting Information

S1 Fig The diagnostic plots of the fitted model in the study area. (TIFF)
