# Supplementary figures and images for: Effects of Climate and Rodent Factors on Hemorrhagic Fever with Renal Syndrome in Chongqing, China, 1997–2008
Source: PLoS One. 2015 Jul 20;10(7):e0133218. doi: 10.1371/journal.pone.0133218 (PMC4507865; doi:10.1371/journal.pone.0133218)

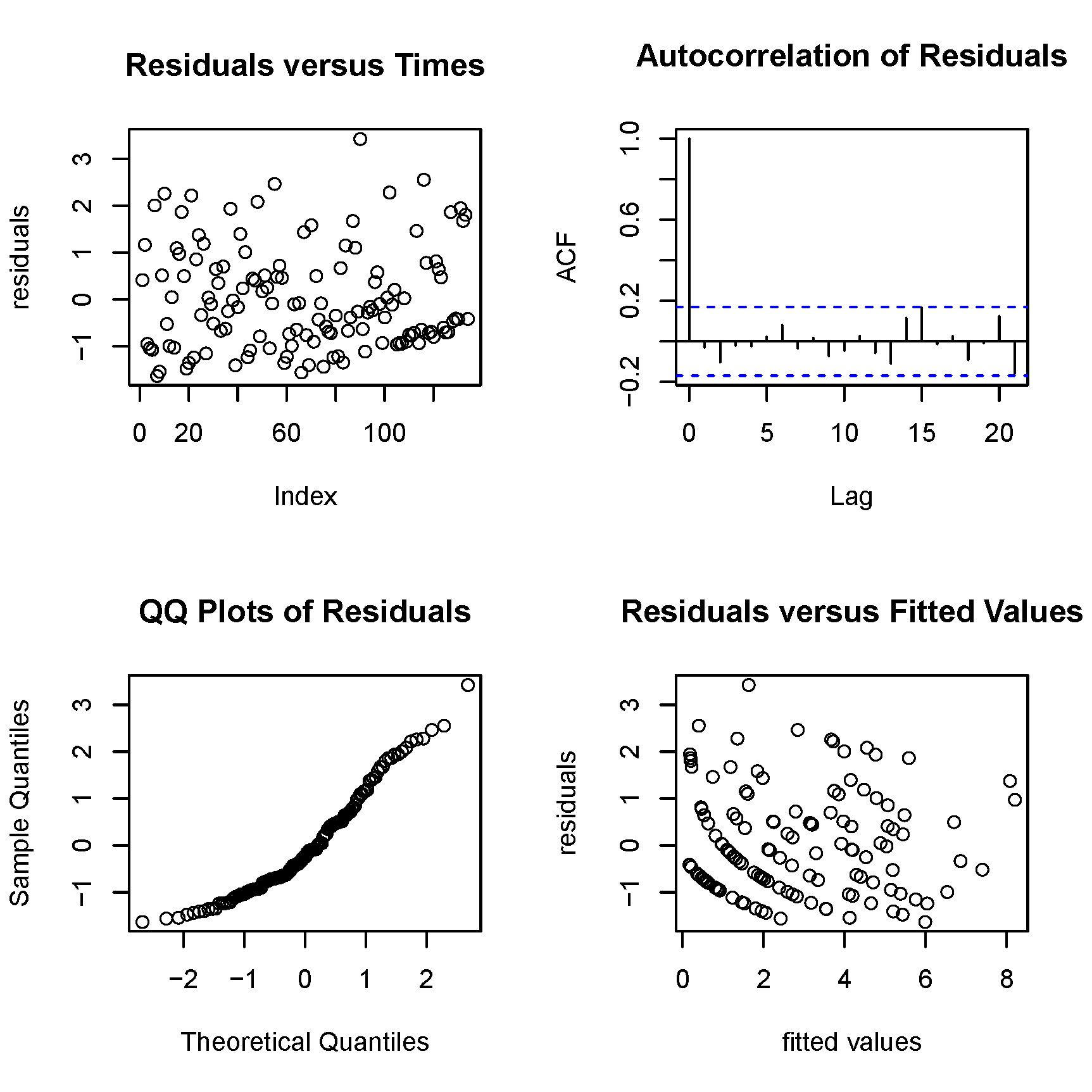

Supplement: S1 Fig — (TIFF) [file pone.0133218.s001.tiff]
